# Impact of tissue heterogeneity corrections in stereotactic body radiation therapy treatment plans for lung cancer

**DOI:** 10.4103/0971-6203.62133

**Published:** 2010

**Authors:** Tania De La Fuente Herman, Heather Gabrish, Terence S. Herman, Maria T. Vlachaki, Salahuddin Ahmad

**Affiliations:** Department of Radiation Oncology, the University of Oklahoma Health Sciences Center, Oklahoma City, OK. USA; 1 British Columbia Cancer Agency, Victoria, BC, Canada

**Keywords:** Heterogeneity corrections, NSCLC, SBRT

## Abstract

This study aims at evaluating the impact of tissue heterogeneity corrections on dosimetry of stereotactic body radiation therapy treatment plans. Four-dimensional computed tomography data from 15 low stage non-small cell lung cancer patients was used. Treatment planning and dose calculations were done using pencil beam convolution algorithm of Varian Eclipse system with Modified Batho Power Law for tissue heterogeneity. Patient plans were generated with 6 MV co-planar non-opposing four to six field beams optimized with tissue heterogeneity corrections to deliver a prescribed dose of 60 Gy in three fractions to at least 95% of the planning target volume, keeping spinal cord dose <10 Gy. The same plans were then regenerated without heterogeneity correction by recalculating previously optimized treatment plans keeping identical beam arrangements, field fluences and monitor units. Compared with heterogeneity corrected plans, the non-corrected plans had lower average minimum, mean, and maximum tumor doses by 13%, 8%, and 6% respectively. The results indicate that tissue heterogeneity is an important determinant of dosimetric optimization of SBRT plans.

## Introduction

As the therapeutic radiation beam traverses the patient’s body, it interacts with tissues of different densities. The conventional method to predict dose to a patient is to assume patient’s body as a homogeneous water medium. Objects of different densities (tissue heterogeneities) are then accounted for analytically with improved algorithms to reduce uncertainties in absolute dose. The dose is thus calculated in a water equivalent material and multiplied by a tissue heterogeneity correction factor. The correction factor is generated from an electron density matrix derived from a CT value matrix since CT value and electron density has a linear relationship. To obtain a correction factor, the present study utilized the tissue heterogeneity correction algorithm known as “Modified Batho Power Law (MBPL)” available in Eclipse Treatment Planning System (Varian Medical Systems, Palo Alto, CA). Batho has been an empirical correction factor method, which uses tissue maximum ratios (TMR), rose to a power that depends on the medium’s electron density relative to water. It was originally developed for dose calculations in water below a single slab of lung tissue.[1] MBPL method differs from the original Batho in its definition of depth. In high energy photon beams the buildup region can be several centimeters thick in which the TMR values are not valid. The MBPL method uses only the descending part of the TMR curves by adding to the depth, the depth of maximum dose.

The dosimetric impact of heterogeneity corrections in Stereotactic Body Radiation Therapy (SBRT) treatment plans, in which an ablative dose, delivered in few fractions to treat medically inoperable patients with early stage Non-Small-Cell Lung Cancer (NSCLC) was studied. The identifying information pertaining to all the subjects of this study was erased to preserve their anonymity. An Institutional Review Board (IRB) clearance was obtained.

## Materials and Methods

### Patients

**Figure 1:**
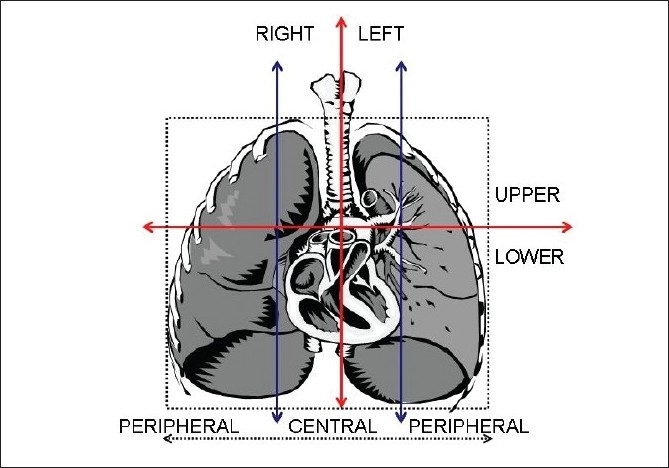
Lung tumor site and location classification criteria

Fifteen low stage NSCLC patients were treated with SBRT at the department of radiation oncology at the University of Oklahoma Health Sciences Center (OUHSC). The four-dimensional computed tomography (4D CT) data from these patients was retrieved and saved to generate plans for this study. All patients were male and the group average age was 69.3 years (range 58 to 85 years). The sites and locations of the tumors were also classified by dividing the chest cavity in the coronal orientation into three equal portions (peripheral right, central, and peripheral left) at the level of the carina, and the thorax was segmented by a transversal line at the carina to divide the upper from the lower lung regions [Figure 1]. The tumor location was categorized as “central” if only tumor margin touched the line defining the central region. There were six tumors in the right-upper-peripheral region (RUP), two in the left-lower-peripheral (LLP), four in the left-upper-peripheral (LUP), two in the right-lower peripheral region (RLP), and one in the left-upper-central (LUC).

### Treatment plan

**Figure 2:**
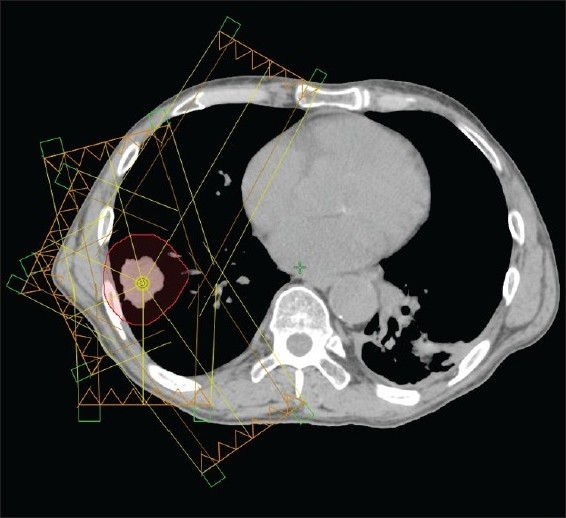
A typical six field treatment configuration showing beam direction towards tumor.

Patients were scanned with a GE Light Speed CT scanner and the Real-Time Position Management, RPM Gating System ver.1.6.5 by Varian Medical Systems, Palo Alto, CA. The CT images were sorted using the 4D planning software (GE Advantage Workstation aw4.3_06) and SBRT treatment plans were generated using Eclipse Treatment planning system. The plans for this study used a prescription dose of 60 Gy in three fractions (20 Gy/fraction). All plans had 4 to 6, 6 MV co-planar non-opposing photon beams as shown in Figure 2 optimized with tissue heterogeneity corrections for patient treatment. The standard dose calculation algorithm pencil beam convolution (PBC) with MBPL for tissue heterogeneity was used. All plans were normalized to deliver the prescribed dose to at least 95% of the planning target volume (PTV) keeping the dose to the spinal cord under 10 Gy. No other normal tissue constraints were specified.

A new set of SBRT treatment plans for all 15 patients was then generated by recalculating, without heterogeneity correction, the previously optimized SBRT plans used for treatment while keeping all beam arrangements (for example: gantry angle, field size, field fluences, monitor unit etc.) identical. These new plans were then compared with plans generated earlier using heterogeneity corrections.

### Statistical analysis

The statistical comparison between the plans with and without tissue heterogeneity corrections was performed using a two-tail t-test and tabulated using a Microsoft Excel spreadsheet. A *P*-value < 0.05 was considered statistically significant.

## Results

The average planning target volume (PTV) was 46.4 cc (range, 2.8 cc to 137.2 cc). The minimum, mean and maximum PTV doses for all patients derived from the heterogeneity corrected and non-corrected homogeneous treatment plans are shown in Table 1. Relative to the heterogeneity corrected treatment plans, the non-corrected treatment plans had average minimum, mean, and maximum PTV doses reduced by 13%, 8%, and 6% respectively (all *P*-values < 0.0001).

**Table 1:** Patient data classified by tumor site and location

*Patient*	*Tumor site and position*	*Plans with tissue heterogeneity corrections*				*Plans without tissue heterogeneity corrections*			
		*%PTV volume receiving prescribed dose*	*minimum dose (Gy)*	*mean dose (Gy)*	*maximum dose (Gy)*	*%PTV volume receiving prescribed dose*	*minimum dose (Gy)*	*mean dose (Gy)*	*maximum dose (Gy)*
1	RUP	95.20	56.71	62.70	66.89	61.00	47.58	58.53	64.59
8		95.00	56.85	62.48	65.54	0.00	48.17	54.54	57.98
10		95.50	56.11	63.08	66.74	35.70	48.84	58.43	63.64
11		95.10	57.13	63.20	67.79	56.10	49.82	60.15	66.17
14		95.80	56.83	63.64	67.35	28.70	51.89	58.52	62.97
15		95.20	56.74	62.75	67.00	27.60	49.72	57.97	62.91
5	LUP	99.00	58.93	63.34	67.12	38.10	51.84	58.88	63.19
7		95.10	57.29	62.72	67.04	34.50	48.50	57.92	62.74
9		95.50	56.61	62.58	65.53	26.00	49.46	57.83	62.91
12		95.50	58.30	62.85	68.21	70.20	55.34	61.37	66.96
4	LUC	95.20	57.03	62.72	65.92	16.10	51.90	58.11	61.59
6	RLP	95.00	56.63	63.19	68.38	23.60	50.37	57.88	63.11
13		95.10	58.06	62.44	66.57	34.70	51.33	58.63	63.16
2	LLP	95.70	57.20	62.63	66.70	5.30	46.45	55.64	61.46
3		95.00	58.43	62.27	64.71	0.00	50.47	55.04	57.74

(RUP (right-upper-peripheral), LUP (left-upper-peripheral), LUC (left-upper central), RLP (right-lower peripheral), and LLP (left-lower-central)). The percentages of PTV volume receiving the prescription dose, and minimum, mean, and maximum PTV doses are presented for plans calculated with and without tissue heterogeneity

**Figure 3:**
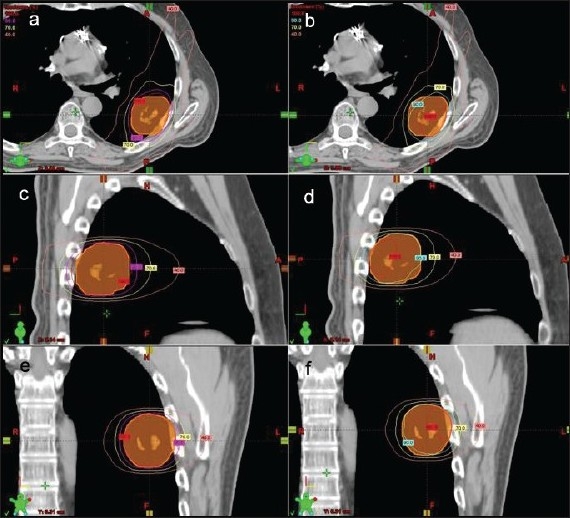
Transverse views of isodose distribution curves derived from heterogeneity corrected (a) and non-corrected plans (b); sagittal views from heterogeneity corrected (c) and non-corrected plans (d); and coronal views from heterogeneity corrected (e) and non-corrected plans (f) of a typical non-small cell lung cancer patient

The percentage of PTV volume receiving the prescription dose for both tissue heterogeneity corrected and non-corrected plans are also shown in Table 1. During planning, the heterogeneity corrected plans were optimized to restrict the percentage of PTV volume receiving dose below prescription to no more than 5%. The percentage of PTV volume not receiving the prescribed dose in the non-corrected plans was very large (≥ 30%) (P-value < 0.0001). Figure 3 shows pictures of isodose distribution curves derived from heterogeneity corrected and non-corrected plans in transverse, sagittal and coronal views of a typical lung cancer patient.

**Figure 4:**
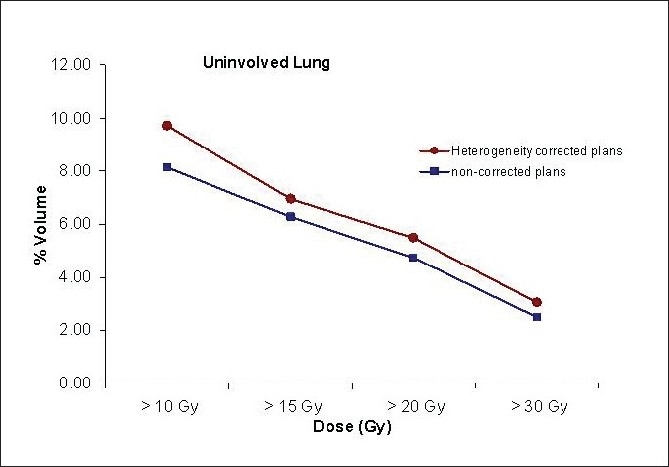
Percentage of uninvolved lung volume receiving doses higher than 10 Gy, 15 Gy, 20 Gy, and 30 Gy for tissue heterogeneity corrected and non-corrected plans.

The average percentage of uninvolved lung volume receiving minimum doses of 10 Gy (V_10Gy_), 15 Gy (V_15Gy_), 20 Gy (V_20Gy_) and 30 Gy (V_30Gy_), was below 10%, 7%, 6%, and 4% respectively for both groups, and the non-corrected plans resulted in slightly less dose to the normal lung, all shown in Figure 4 (all *P*-values < 0.0001). The average mean and maximum doses to the normal lung were 3.5 Gy and 64.0 Gy for the corrected and 3.0 Gy and 60.6 Gy for the non-corrected plans respectively (*P*-values < 0.0001). The average maximum spinal cord dose was 8.6 and 9.7 Gy for corrected and non-corrected plans, respectively (no statistical significance, *P*-value = 0.7).

## Discussion

Stereotactic Body Radiation Therapy (SBRT) for medically inoperable Stage I lung cancer is an extremely promising technique that has emerged due to recent advancements in technology. Since SBRT delivers a high ablative dose to the target in just a few fractions, it requires accurate treatment planning with a precise radiation delivery system. Studies are currently being conducted to understand the effect of different heterogeneity correction methods on dose distribution, so that dose-volume relationships which determine the likelihood of tumor control as well as acute and long term side effects can more accurately be determined.[2–5]

Chang *et al*.[2] studied the impact of heterogeneity correction on dosimetric parameters of V_20_ or Mean Lung Dose (MLD) that predict radiation pneumonitis, and concluded that, “a high degree of correlation exists between heterogeneity-corrected and heterogeneity-uncorrected dosimetric parameters for lung and the risk of developing pneumonitis”. Kong *et al*.[3] also reported results for radiation induced toxicities in a dose escalated study for patients with NSCLC based on treatment plans that were corrected using equivalent path length algorithms like Batho. The investigators found that a V_20_ of 30% and 20 Gy or greater for the MLD were predictors of lung toxicity.

Ding *et al*.[4] investigated the influence of heterogeneity corrections on tumor and normal lung dosimetry in SBRT for lung cancer treatment. They compared treatment plans with heterogeneity corrections using the path length correction in a pencil beam algorithm and treatment plans with same beam arrangements and monitor units without heterogeneity corrections. The prescribed doses for both plans were 48 Gy to 60 Gy in three fractions. They quantified the tumor dose difference between both plans using equivalent uniform doses (EUDs). The study reported that without tissue heterogeneity corrections, the plans provided much lower EUD doses to the tumor, and the doses to normal lung were also significantly reduced.[5] The calculated average EUD difference between both plans was 15.1%. Similar to that study, the results found in our study indicate a PTV dose reduction (13%, 8% and 6%, respectively on average minimum, mean, and maximum doses) when the treatment plans were optimized with heterogeneity corrections and then recalculated without heterogeneity corrections keeping same beam arrangements and monitor units.

In this study, we have used PBC photon beam dose calculation algorithm with MBPL heterogeneity corrections for dose instead of scattered based collapsed cone convolution (CCC) or anisotropic analytical algorithm (AAA) which achieves increased accuracy in scattered dose calculation. A recent Japanese study[6] reported a discrepancy in dose of about 2% calculated by PBC with MBPL compared to that calculated by AAA in stereotactic lung irradiation. A prescription dose reduction from 20 Gy per fraction to 18 Gy per fraction in three fractions is now suggested by the quality assurance working group of the phase III Rosel study[7] when utilizing AAA or CCC dose calculation models instead of PBC with MBPL.

## Conclusions

Lung tissue filled with air is significantly less dense than other body tissues, the failure to use heterogeneity corrections creates plans which overdose the target because the planning system optimizes beam paths assuming more attenuation than actually occurs. This discrepancy also alters the optimization of beam angles as well as monitor units. The more conformal the treatment, and the higher the dose per fraction, the more critical is the use of this correction, because while homogeneous plans look good on the computer screen, in reality, the dose coverage of the PTV will often be poor enough to significantly decrease the tumor control probability.
